# The Impact of Funding through the RF President’s Grants for Young Scientists (the field – *Medicine*) on Research Productivity: A Quasi-Experimental Study and a Brief Systematic Review

**DOI:** 10.1371/journal.pone.0086969

**Published:** 2014-01-27

**Authors:** Ruslan T. Saygitov

**Affiliations:** Independent researcher, Moscow, Russia; Max Planck Society, Germany

## Abstract

The impact of grants on research productivity has been investigated by a number of retrospective studies. The results of these studies vary considerably. The objective of my study was to investigate the impact of funding through the RF President’s grants for young scientists on the research productivity of awarded applicants. The study compared the number of total articles and citations for awarded and rejected applicants who in 2007 took part in competitions for young candidates of science (CoS’s) and doctors of science (DoS’s) in the scientific field of medicine. The bibliometric analysis was conducted for the period from 2003 to 2012 (five years before and after the competition). The source of bibliometric data is the eLIBRARY.RU database. The impact of grants on the research productivity of Russian young scientists was assessed using the meta-analytical approach based on data from quasi-experimental studies conducted in other countries. The competition featured 149 CoS’s and 41 DoS’s, out of which 24 (16%) and 22 (54%) applicants, respectively, obtained funding. No difference in the number of total articles and citations at baseline, as well as in 2008–2012, for awarded and rejected applicants was found. The combination of data from the Russian study and other quasi-experimental studies (6 studies, 10 competitions) revealed a small treatment effect – an increase in the total number of publications over a 4–5-year period after the competition by 1.23 (95% CI 0.48–1.97). However, the relationship between the number of total publications published by applicants before and after the competition revealed that this treatment effect is an effect of the “maturation” of scientists with a high baseline publication activity – not of grant funding.

## Introduction

Grants (public or private) is one of the sources of funds for research. Today the growing number of foundations that distribute research grants reveals that grants are an efficient way of investing funds in science. This idea is substantiated by the findings of numerous retrospective studies which point to an increase in the number of articles published by awarded applicants [1], [2], [3], [4], [5], [6], [7], the number of times articles were cited [4], [5], as well as patents [8]. Analyses of the effect of grants in subgroups also quite often indicated the advantageousness of grant funding for young scientists [1], [7], [9], [10]. However, the correction of the findings of some of these studies based on baseline differences between groups of awarded and rejected applicants shows the link between grants and research productivity to be weaker [5], [6], [7] or totally erases it [3]. Moreover, several studies even revealed a negative effect of grants on the research productivity of awarded applicants [10], [11], [12]. Inconsistencies of the studies result, along with the unavailability of randomized studies, have put a question mark over research grant funding being effective.

In Russia, the system of scientist grant support was introduced in 1992 along with the foundation of the Russian Foundation for Basic Research (RFBR) [13]. Later on, in 2003, the first RF President’s grants were issued to young scientists – to candidates (CoS) and doctors (DoS) separately (for more detail about awarding of academic degrees in Russia see Table S1). Grants were issued (as is the current practice) for a two-year period to finance basic and applied research studies in the priority areas of Russian science, technology, and engineering (a total of nine areas). Over the period of time these foundations were in existence, considerable funds had been allocated for research and academic projects. For instance, RFBR competitions – the major source of grant funding for research activity in Russia – received over 5 billion rubles in 2007, and already 8 billion in 2012 (http://www.rfbr.ru/rffi/ru/funding). That said, the issues of how effectively these funds were distributed (i.e. whether funds were issued to truly the best researchers for the best projects) and their impact on research productivity have not been studied up until now.

### Objective

To investigate the impact of funding through the RF President’s grants for young scientists – CoS’s and DoS’s – on the research productivity of awarded applicants.

## Methods

### Competition Participants

The list of all the participants (awarded and rejected applicants) in the competition organized by the Council for the RF President’s Grants for Young Scientists was available for the year 2007 only. The list of all the applicants with the CoS degree (age ≤35 years), the topics chosen by the applicants, and the institutions they were full-time employees of, is available on http://grants.extech.ru/spisok_kand_2007.php. The list of the awarded applicants is posted on http://grants.extech.ru/grants/res/winners.php?OZ=7&TZ=K&year=2007. The list of all the applicants with a DoS degree (age ≤40 years) is available on http://grants.extech.ru/spisok_dok_2007.php, and the list of awarded applicants – on http://grants.extech.ru/grants/res/winners.php?OZ=7&TZ=D&year=2007. Applications (young scientists participated as principal investigators) had been accepted in December 2006. The results of the competition were announced in April 2007. The funding of awarded applicants started in the second half of 2007. The funding volume over two years was 250 thousand rubles for CoS’s and 500 thousand rubles for DoS’s (http://grants.extech.ru/izv_k2007.php) or around 10 and 20 thousand US dollars, respectively, based on the exchange rate as of September 2007. The study uses data for applicants who participated in the competition in the field of medicine.

### Bibliometric Analysis

The impact of grant funding was assessed based on the number of total articles per person and their citation counts in 2008–2012 (ex-post). Data on the number of total articles and their citation counts over the period of 2003 to 2007 (ex-ante) was taken into account with a view to ensuring control over publication and citation rates in 2008–2012 based on baseline data.

This study’s data on the number of total articles per person and total citation counts per person is taken from the eLIBRARY.RU database (for more detail on the database see Table S2). The search was carried out between 15.03.2013 and 17.04.2013. The information on applicants’ publication and citation was primarily taken from pages containing the authors’ personal profiles (the “Author Index” section). In the event there was no information on articles published or no applicant’s personal profile available, an additional search was performed using the eLIBRARY.RU search system (the “Full-text Search”). The system’s capabilities make it possible to specify search queries using not only the last name but the full name and patronymic of the author. If need be, search queries can be specified using the author’s specialty and a necessary time interval. All this makes it possible to almost entirely forestall the use of homonyms’ bibliometric data. In one-off cases, in the event homonyms were detected (there was a last name and initials match), the list of publications and their citations was specified taking account of the applicant’s place of employment (the information is provided in both the database for the competition participants and the eLIBRARY.RU database).

In calculating the number of total articles and citation counts, the study considered any type of scientific articles (original articles, reviews, case reports, etc.). The analysis excluded articles that did not contain the results of research or original scientific analytics (including those dedicated to scientist and scientific society anniversaries, reports on scientific events, editorials, book reviews, letters, notes, etc.). To count as an article, the publication was to be accompanied with a resume and/or a reference list. The bibliometric analysis did not include meeting (or congresses, conferences, and other professional gatherings) abstracts, books or chapters.

### The Defense of Doctoral Dissertations

The study registered cases of the defense of doctoral dissertations by applicants with the CoS degree at baseline (as of the time applications for the RF President’s Grants competition had been submitted). The data was analyzed for the period of late 2006 through 2012. Information on the dates doctoral dissertations were defended was received in mid-November 2012 from the official website of the national government agency *The Vysshaya Attestatsionnaya Komissiya* (“Higher Attestation Commission”) or the *VAK* (http://vak.ed.gov.ru/ru/dissertation/). This agency oversees the awarding of advanced academic degrees. Posting an announcement on the doctoral dissertation defense on the *VAK* website is a mandatory condition of defense. A brief description of the procedure for the defense of a doctoral dissertation is provided in Table S1. The defense of doctoral dissertations by applicants with the CoS degree after submitting an application to the competition can be regarded as a qualitative criterion for the impact of grant funding on the research productivity of this group of young scientists.

### Statistical Analysis

The statistical analysis was performed using SPSS v. 15.0 (SPSS Inc). Independent group (awarded and rejected applicants) differences were assessed using the Manne-Whitney U test (for continuous and count data) and the chi-squared test (for categorical variables). The comparison of two related samples (count data before and after competitions) was performed using the Wilcoxon test. To assess the relation between the independent variable “applicants” (where 0 is rejected, and 1 is awarded applicants) and the dependent variables “ex-post number of total articles” and “ex-post total citation counts” (both count data), negative binomial regression models were applied. The correction of this relation was done taking account of baseline factors (gender, city, place of employment, and specialty) and covariates (the number of total articles and citations before competitions and the rank of the current institution).

The fraction of applicants with the CoS degree who defended a doctoral dissertation after applying to the competition was assessed using Kaplan-Meier curves, and the log-rank (Mantel-Cox) test was used to compare the “survival” distributions of the two samples. The probability of defending a doctoral dissertation with an adjustment for baseline group differences was assessed using the Cox proportional hazards model. The impact of the independent variable on the probability of the outcome was determined using the odds ratio and 95% confidence interval (CI).

## Results

### Characteristics of Applicants

The 2007 competition featured 149 CoS’s and 41 DoS’s from 79 research and academic institutions. Respectively, 24 (16%) and 22 (54%) applicants came out winners. Females made up over half of the applicants who participated in the competition for young CoS’s and over 40% of those who took part in the competition for young DoS’s (Table 1). A fourth of all the applicants worked in Moscow and Saint Petersburg; yet in the group of awarded applicants the share of such scientists was higher (which has been substantiated with regard to participants in the competition for young CoS’s). On the whole, the two contests combined featured works across 46 biomedical specialties. Proposal topics had the signs of multidisciplinary projects (2–4 specialties) with 85 (68%) rejected and 14 (58%) awarded applicants with the CoS degree (p = 0.358) and with 8 (42%) and 14 (64%) rejected and awarded applicants with the DoS degree (p = 0.168). Awarded applicants with the CoS degree were more frequently engaged in oncology, but not immunology, than their counterparts.

**Table 1:** The characteristics of awarded and rejected applicants with the CoS or DoS degree.

Category	Subcategory	CoS’s group		p	DoS’s group		p
		Rejected,n = 125	Awarded, n = 24		Rejected,n = 19	Awarded,n = 22	
Sex, abs. (%)	Female	70 (56)	13 (54)	1.000	8 (42)	9 (41)	1.000
City, abs. (%)	Moscow	14 (11)	12 (50)	(df = 2) 0.001	3 (16)	6 (27)	(df = 2) 0.231
	Saint Petersburg	5 (4)	4 (9)		0	2 (9)	
	Other	106 (85)	24 (52)		16 (84)	14 (64)	
Institution type^1^, abs. (%)	Research institute	42 (34)	12 (50)	(df = 2) 0.275	0	4 (18)	(df = 2) 0.09
	University	81 (65)	12 (50)		18 (95)	18 (82)	
	Other	2 (2)	0		1 (5)	0	
Academy of Sciencesinstitution^2^, abs. (%)		27 (22)	5 (21)	0.739	0	2 (9)	0.490
Specialties^3^, abs. (%)	Immunology	24 (19)	0	0.014	1 (5)	0	0.463
	Oncology	13 (10)	7 (29)	0.032	1 (5)	4 (18)	0.350
	Physiology	15 (12)	1 (4)	0.471	4 (21)	4 (18)	1.000
	Pediatrics	14 (11)	2 (8)	1.000	1 (5)	7 (32)	0.05
	Genetics	14 (11)	6 (25)	0.136	1 (5)	2 (9)	1.000
	Cardiology	15 (12)	3 (13)	1.000	2 (11)	4 (18)	0.668
Rank of current institution^4^		333 (189; 455) (n = 122)	187 (90; 358) (n = 23)	0.089	167 (57; 351) (n = 18)	252 (142; 351) (n = 22)	0.325

**Note**: ^1^Work at a university differs from that at a research institute, mainly, in the need to complement one’s scientific activity with teaching.

2 An Russian Academy of Sciences or an Russian Academy of Medical Sciences institution.

3 The specialty was determined based on the topic of the proposal in conformity with the RF-maintained list of specialties for scientists (http://vak.ed.gov.ru/ru/help_desk/). The table lists the most common specialties (with the number of applicants ≥10% of the total number contestants). Any single proposal could contain ≥2 specialties.

4 Positions in the ratings were presented as median (25^th^ and 75^th^ percentiles). This rating is calculated given the total number of publication of an institution’s employees over the period of 2007 to 2011 (data is available on http://elibrary.ru). The highest position in the ratings for institutions listed in eLIBRARY.RU database, is 1, and the lowest –1812; 5 institutions wherein applicants worked full-time are not included in the ratings (the data was not available in the eLIBRARY.RU database in early 2013).

### Bibliometric Data

The *Author Index* section of the eLIBRARY.RU database contained data on the publication activity of 134 (71%) of 190 applicants. For the rest, the search for articles and their citation was performed using the eLIBRARY.RU search system. As a result, I found references to 4327 works published in 2003–2012 and marked as “articles” in the database. The analysis of brief descriptions for these materials revealed that only 3529 (81.6%) publications were original scientific articles. In other cases, the eLIBRARY.RU database contained references to meeting abstracts, duplicate publications, as well as publications that did not contain research study results (for more detail see METHODS: Bibliometric analysis).

**Figure 1:**
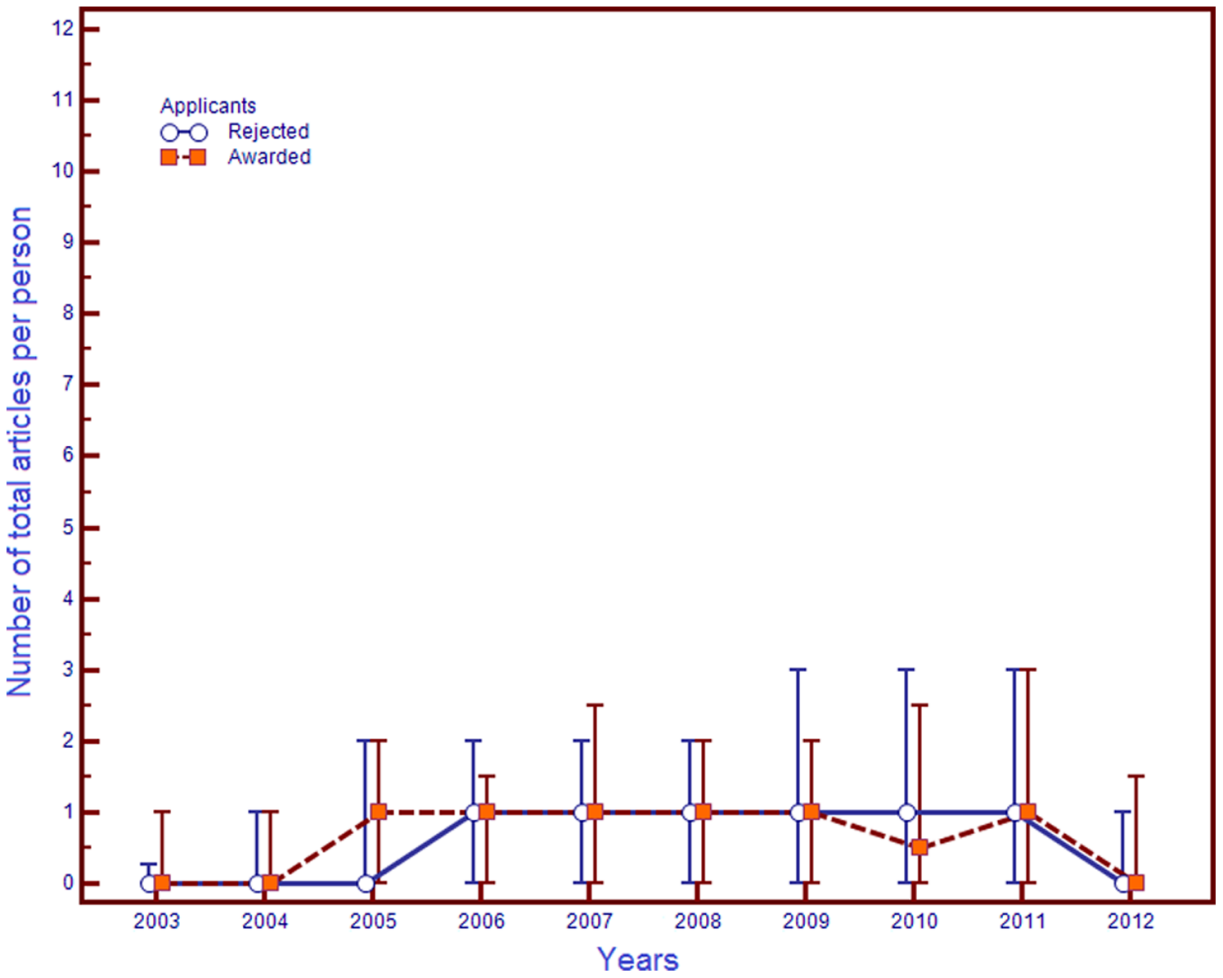
Trends for the number of total articles published by young CoS’s in 2003–2012. **Note.** Data were presented as median (markers), 25 and 75 percentiles (error bar). The figure is drawn using MedCalc Statistical Software v. 12.7.5 (MedCalc Software bvba, Ostend, Belgium).

**Figure 2:**
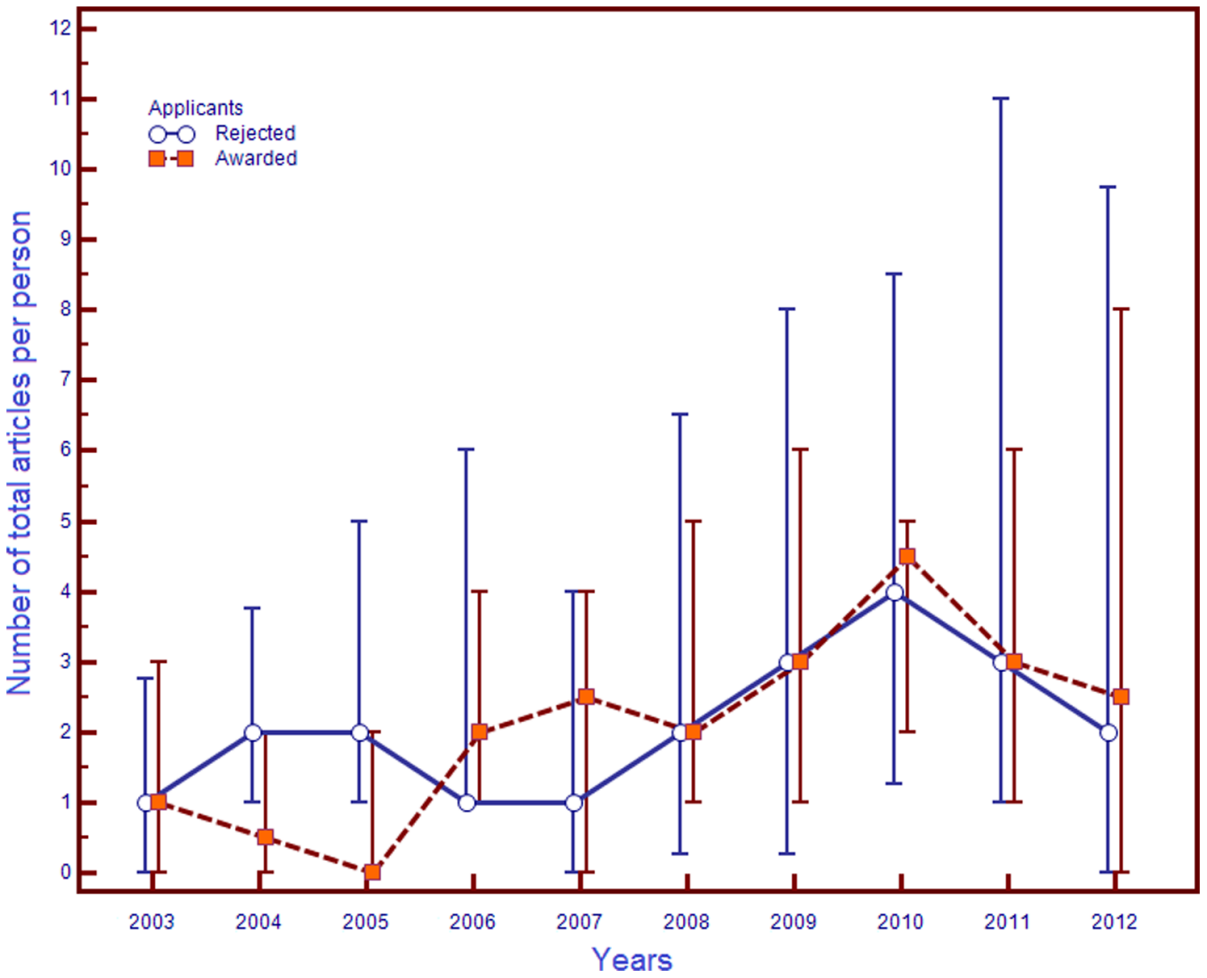
Trends for the number of total articles published by young DoS’s in 2003–2012. **Note.** Data were presented as median (markers), 25 and 75 percentiles (error bar). The figure is drawn using MedCalc Statistical Software v. 12.7.5 (MedCalc Software bvba, Ostend, Belgium).

The number of total articles per person and total citations per person for awarded and rejected applicants, young CoS’s and DoS’s, did not differ both before and after competitions (Table 2). Does that mean that the publication activity of applicants and the citability of their articles did not change after the competition? The comparison of paired (before-after) samples revealed unidirectional changes – particularly, an increase in the number of total articles (except for awarded applicants with the CoS degree) and their citations. The analysis of the yearly publication activity of young scientists revealed that the median number of articles published by applicants with the CoS degree did not change (in average) for a long time (Fig. 1). On the contrary, with applicants with the DoS degree, the yearly increase in the number of articles published reached its peak in the third year after the competition – with both awarded and rejected applicants (Fig. 2).

**Table 2:** The median number of total articles and their citations for awarded and rejected applicants before (2003–2007 years) and after (2008–2012 years) competitions.

Category	Subcategory	CoS’s group		p*	DoS’s group		p*
		Rejected, n = 125	Awarded, n = 24		Rejected, n = 19	Awarded, n = 22	
**Articles**	2003–2007 years	3 (1; 7)	4 (2; 8)	0.284	9 (3; 21)	8.5 (3; 17.5)	0.479
	2008–2012 years	6 (2; 11.5)	5.5 (1; 11)	0.808	15 (6; 44)	16 (10; 25)	1.000
	Δ (ex-post – ex-ante)	2 (−0.5; 6.5)	0 (−1; 4)	0.147	4 (−2; 19)	6.5 (2; 16)	0.734
**p** **		0.001	0.191		0.012	0.001	
**Citations**	2003–2007 years	0 (0; 3)	1.5 (0; 4)	0.117	2 (1; 5)	3 (1; 8)	0.493
	2008–2012 years	2 (0; 7.5)	3 (1; 7.5)	0.096	4 (1; 31)	6.5 (2; 13)	0.704
	Δ (ex-post – ex-ante)	1 (0; 4)	1.5 (−1; 5.5)	0.873	2 (−1; 19)	2 (0.5; 8)	0.854
**p** **		0.001	0.012		0.016	0.009	

**Note:** Count data and values of delta (Δ) were presented as median (25^th^ and 75^th^ percentiles).

* the p values were calculated based on the results of the comparison of independent groups (awarded and rejected applicants) using the Manne-Whitney U test;

** the p values were calculated based on the results of pair comparisons (before and after the competition) using the Wilcoxon test.

In the negative binomial regression models, no statistically significant differences between awarded and rejected applicants in the number of ex-post total articles or citations were detected, including after the adjustment for baseline factors (gender, city, place of employment, and specialty) and covariates (the number of ex-ante total articles/citations per person and the rank of the current institution) (Table 3). From the number of factors and covariates included in the regression model only the number of ex-ante total articles was the independent predictor of future publication activity: for CoS’s group model the regression coefficient is 0.097 (standard error 0.023) (p<0.001), for DoS’s group model –0.066 (0.023) (p = 0.004).

**Table 3:** Negative binomial regression models predicting the ex-post number of total articles and their citations for awarded applicants (vs. rejected applicants as reference group).

Variables	CoS’s group			DoS’s group		
	Model 1	Model 2	Model 3	Model 1	Model 2	Model 3
Ex-post number of total articles	−0.088 (0.237)	−0.173 (0.242)	−0.022 (0.305)	−0.309 (0.320)	0.118 (0.335)	0.346 (0.441)
Ex-post total citations counts	0.144 (0.235)	0.439 (0.247)	0.412 (0.324)	−0.409 (0.323)	0.422 (0.363)	0.442 (0.480)

**Note**. The link between dependent variables (“ex-post number of total articles” and “ex-post total citation counts”) and independent variables is presented as regression coefficients (standard error). Model 1 – an independent variable “applicants” only (where 0 stands for rejected and 1 for awarded applicants); Model 2 – Model 1 + “ex-ante number of total articles” and “ex-ante total citation counts”: Model 3 – Model 2 + all factors from Table 1 and covariate “Rank of current institution”. The category “immunology” of the variable “specialty” was not taken into account in the regression analysis due to the absence of awarded applicants with this specialty. Five missing values of the variable “rank of current institution” are replaced with the maximum values of the attribute [rank = 1486] in the sample. Thus, these institutions were given low positions in the eLIBRARY.RU institute ratings (the argument – the position in the ratings is not assessed for institutions whose employees publish few articles). No statistically significant link between the independent variable “applicants” and the dependent variables “ex-post number of total articles” and “ex-post total citation counts” was detected in any of the regression models (in all of the cases p>0.05).

### The Defense of Doctoral Dissertations after Competitions (for CoS’s Applicants)

**Figure 3:**
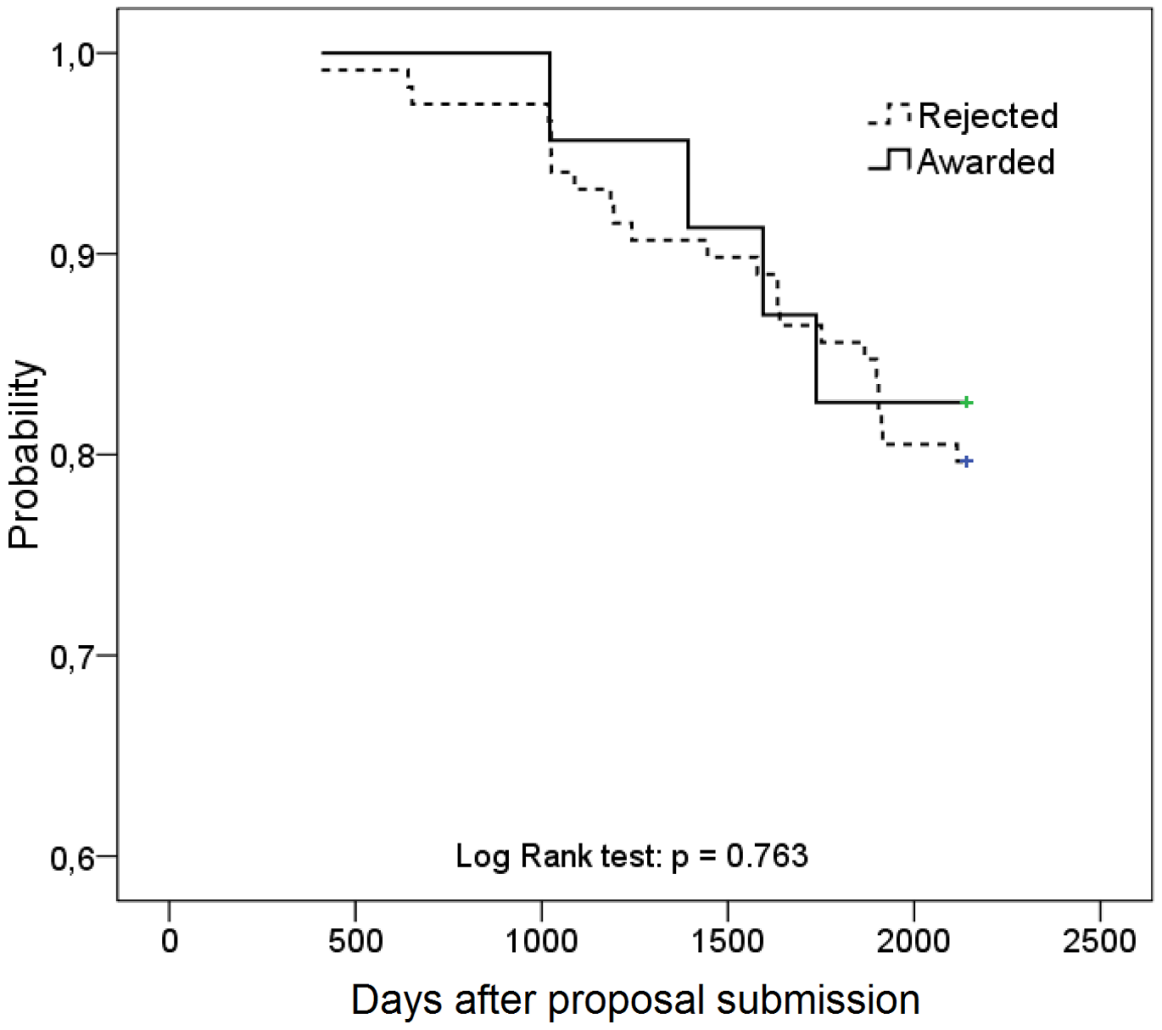
The time-related (2008–2012) probability of the doctoral dissertation defense by CoS applicants.

Over the entire period following the submission of applications for grants (from December 2006 to November 2012) there were 5 (21%) awarded applicants and 31 (25%) rejected applicants defending a doctoral dissertation (p = 0.678). Out of this number, in 2008–2012 (i.e. after funding for awarded applicants began) there were 4 (17%) and 24 (20%) applicants defending a doctoral dissertation, respectively (p = 1.000; Fig. 3). The time-related probability of defending a doctoral dissertation in 2008–2012 for the group of awarded applicants compared with that of rejected applicants was 0.85 (95% CI 0.30–2.45). With an adjustment for such dummy variables as “city” (1– “Moscow/Saint Petersburg”, 0– “another city”), “specialty” (1– “Oncology”, 0– “another specialty”), “institution’s rating” (1– rank<median [<331], 0–≥ median), the number of ex-ante total articles (1–>median [>3], 0–≤ median) and their ex-ante citation counts (1–>median [>0], 0– equal zero), the probability of defending a doctoral dissertation for awarded applicants did not change and was 1.17 (95% CI 0.36–3.80). A significant predictor of defending a doctoral dissertation in 2008–2012 was only the number of ex-ante total articles. In particular, publishing in 2003–2007 over 3 (median) articles was associated with a 2.53 times increase in the probability of defending a doctoral dissertation (95% CI 1.12–5.68).

## Discussion

The study did not reveal any impact of funding through the RF President’s grants on research productivity (the number of total articles published, their citations, the number of dissertations presented for defense in pursuit of the DoS degree). After an adjustment for the number of ex-ante total articles for awarded and rejected applicants, the absence of differences in future research productivity was confirmed. Is this an outcome common to Russian competitions or do we observe the same in other countries as well?

### The Impact of Grants on Research Productivity: a Treatment Effect or Bias?

I must say that there is no direct evidence (i.e. randomized study findings) that grant-funding support of researchers has an impact on their productivity in any form. Note that there have been published the findings of a whole number of retrospective studies on the publication and citation rate for awarded and rejected applicants. Having examined those, I can note that the size of the effect attributed to grants varies considerably from study to study. Thus, the maximum increase in the number of publications (the difference between the pre-post number of publications in the treatment and control groups) I have come across in analyzing literature was presented by Bohmer et al. [2]: awarded applicants (the scientific field – *Physics*) published an average of 6 articles more than rejected applicants (the analysis period – 4 years before and after the competition). A minimal effect (actually – negative) of grant funding support was detected in a study by Langfeldt et al. [10]: awarded applicants (the scientific field – *Engineering Sciences*) published an average of 4 articles fewer (the analysis period – 5 years). The findings of the largest (by the number of applicants included in bibliometric analysis) studies indicate that awarded applicants published an average of one article more (a 7–20% increase in publication productivity) than rejected ones (the scientific field – *Health-Related Research*; the analysis period – 5 years) [6], [7]. The heterogeneity of results was also characteristic of studies wherein in addition to the number of articles published the researcher studied the effect of grants on the citation count. For example, the highest increase in the citation count of articles by awarded applicants was observed in a study by the Danish Agency for Science, Technology, and Innovation (the scientific field – *Medicine*) [5], the highest decrease (once again – a negative effect of grants) – in a study by Bornmann et al. (the scientific field – *Molecular Biology*) [3].

However, as we know, a substantial limitation of non-randomized studies is the presence of bias in their findings. Correction bias in studies on the effectiveness of grant funding by adjustment of the baseline research productivity of applicants [3], [6], [7], the proposal priority score [6], [7], and demographic data [5] in some cases minimized [6], [7] and even totally erased the link between grants and increases in research productivity after the competition [3]. Note that even such a correction result does not let us get rid of bias in the results of a separate study and thus reproduce the inferences of experimental studies [14], [15]. I see the solution in combining the numeric data from several quasi-experimental and observational studies, which were obtained as a result of a systematic review. This approach in some cases enables us to obtain results comparable with the results of randomized studies [16], [17]. For example, MacLehose RR et al., summarizing the results of their study and results of 4 other reviews on the same issue, came to the conclusion that “the limited evidence available does not support the view that effect size estimates from QEO [*quasi-experimental or observational*] studies are systematically biased.” However, at the same time, the authors stated that “… this does not imply that estimates of effect size from QEO studies are ‘usually valid’” [16].

### Grant Funding of Medical Research Projects: Brief Systematic Review and Meta-analytical Estimates

**Figure 4:**
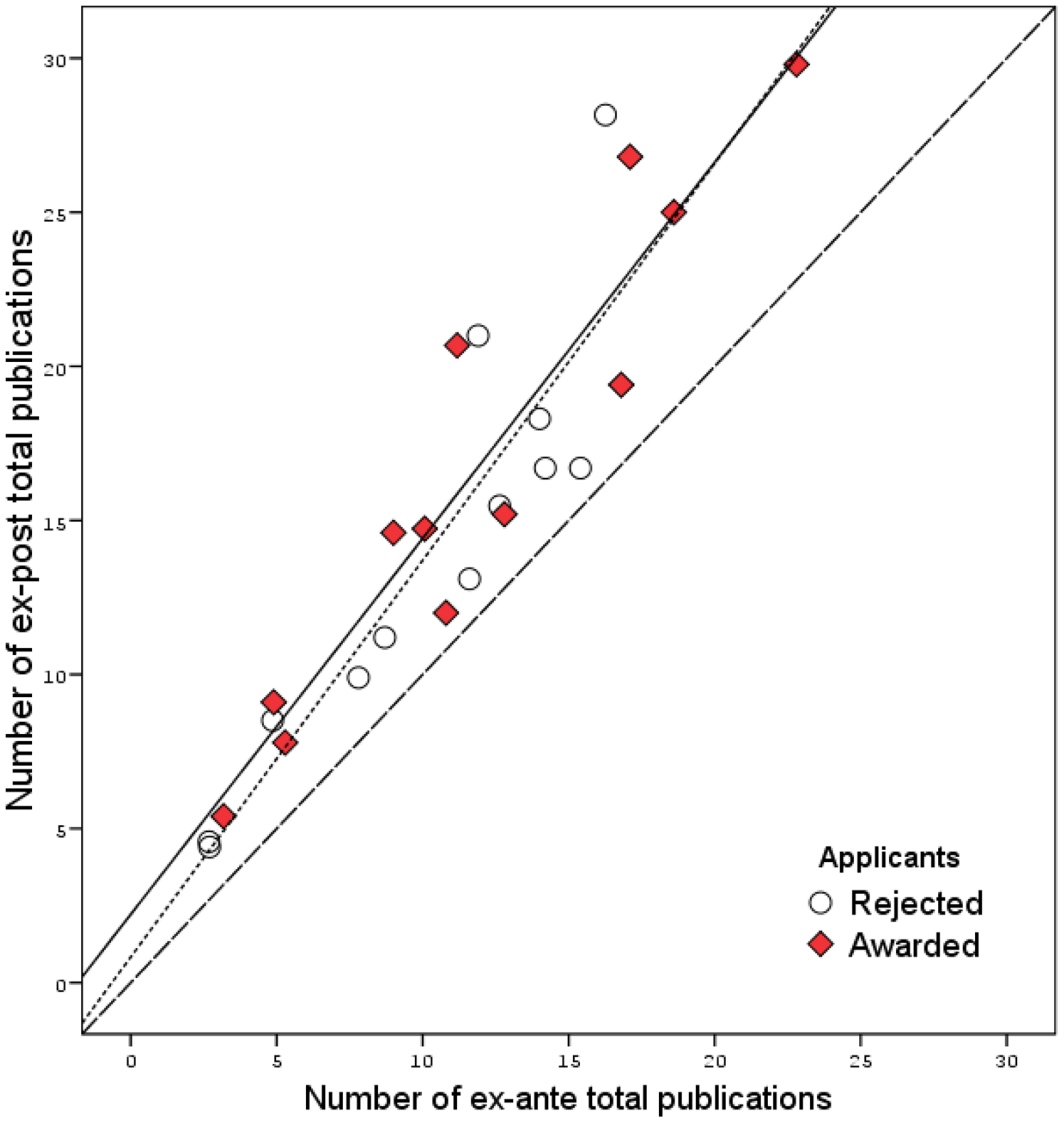
The relationship (pre-post) between the number of total publications of awarded and rejected applicants: the results of seven (including Russian) quasi-experimental studies. **Note.** The dotted line is the regression line for the row of values for the awarded group; the solid line is the regression line for the rejected group; the dashed line is the reference line (the number of total publication ex-ante and ex-post are equal). In the analysis of the number of total publications data from 12 competitions is used (including two Russian).

A systematic review of studies on the impact of grants on the research productivity of awarded applicants has not been carried out by anyone yet. A search let me come across six quasi-experimental studies that examined changes in the research productivity (number of publication and citation count) of awarded and rejected applicants who participated in competitions under the medicine specialty (including clinical medicine, biomedicine, pharmacology/toxicology, social medicine and epidemiology, psychology; for more detail see Table S3, Table S4) [2], [5], [6], [7], [10], [18]. In all of the six studies (data from 10 competitions), changes in the number of total publication of applicants were analyzed, and in two of them [2], [5] – changes in the citation counts. The combination of quantitative data (including data from the Russian study) revealed that the number of articles by awarded applicants had increased (the pooled mean weighted with the study size) from 15.0 to 17.6 over a similar period following the beginning of the funding (4–5 years), and by rejected applicants – from 11.3 to 12.9, respectively (meta-analytical estimations are performed using the StatsDirect statistical package; http://www.statsdirect.com). The summing of a change-from-baseline values of the number of publications and its standard deviation revealed that grant funding was associated with an additional increase in the number of total publications over a 4–5-year period – the pooled effect size (random effects) 1.23 (95% CI 0.48–1.97). However, the heterogeneity of the studies’ results (I^2^ = 90%) does not let us consider the pooled estimate independently. On the other hand, the relationship between the number of total publications ex-post with its level ex-ante indicates that the higher research productivity of applicants prior to the competition, the greater was its increase in the future (Fig. 4). A similar dependency was found for the number of total citations as well (data not shown). Note that this relationship was characteristic of both awarded and rejected applicants. For comparison, exactly the same dependence was also found in comparing the bibliometric data of applicants who participated in 17 FRIPRO competitions (Figure S1) [10]. In my opinion, an increase in the number of total publications ex-post reflects the general trend of the “maturation” of young scientists (in 5 of 6 studies [2], [6], [7], [10], [18] the average age of applicants varied from 32 to 46 years; in the Russian study – <35–40 years; in the Danish study [5] about 40% of applicants were aged up to 45 years), which is expressed in the progressive (until the age of 50) increase in the number of articles published every year [19]–[21]. Note that, judging by the data presented in Figure 4 and Figure S1, the “maturation” of frequently published young scientists (both awarded and rejected) happens faster. These data allow me to put forward a hypothesis on “maturation” bias being the source of a small treatment effect attributed to grants. Note that the impact of bias is the higher, the more researchers with a really high publication number there are in the group of awarded (approximately 10–15 or more articles over a period of 4–5 years of research work; Figure 4, Figure S1). It may seem paradoxical, but it turns out that the weight of “maturation” bias directly depends on the effectiveness of the process of selection of applicants to the competition: the higher it is, the more chances of detecting the bias-based treatment effect we have, and vice versa.

### Limitations

The validity of the eLIBRARY.RU database on articles with results of biomedical researches of Russian scientists has not been studied before. Separate examples point to the incompleteness of this database with regard to both the articles [22] and the citation counts [23]. At the same time, there is no reason to believe that the number of articles and their citations, which are mentioned in the abstract and citation database, varies systematically for awarded and rejected applicants. Besides, according to the developers of eLIBRARY.RU, the database currently contains the same volume of information on international publications by Russian authors as WoS and Scopus [22]. For comparison, the WoS database contained 83% of articles by applicants (published within 4 years before the competition and within no more than 6 years after the competition) who participated in the Emmy Noether Programme competition [2].

In my study, just like in combining data from quasi-experimental studies, I analyzed the number of total articles and citations per applicants. At the same time, the informativeness of these indicators and, in particular, their sensitivity in determining the effect of grant funding can be insufficient. Perhaps, the use of other surrogate markers (for example, the quality-adjusted publications rate, the field-normalized citation rate, or others) would’ve presented different results in assessing the effectiveness of grant funding support [5].

The search for quasi-experimental studies on the effect of grants on the research productivity of awarded applicants who participated in competitions under the medicine specialty was part of a systematic review (data not shown). However, even a systematic review does not rule out publication bias (including time lag bias, grey literature publications). This bias can be the effect of foundation administrations being reluctant to conduct “risky” studies and publish “uncomfortable” data (i.e. data which directly or indirectly attests to the ineffectiveness or little effectiveness of the process of selection of applicants to the competition carried out by the foundation). The effects of publication bias are quite evident – unpublished data can have a substantial impact on the assessment of the size of the treatment effect, especially if the effect is small [24]. However, in comparing effect sizes (difference-in-differences data for number of total publication) and study sizes (the results of 12 competitions, including 2 Russian) no sign of publication bias was found (Figure S2).

## Conclusion

Grants for Russian young scientists did not have an impact on their research productivity. More specifically, after the competition I observed no additional, as compared with the figures for rejected applicants, increase in the number of total articles published, citations, and the number of scientists with the CoS degree who defended a doctoral dissertation. The combination of data from the Russian study and quasi-experimental studies conducted in USA, Germany, Norway, and Denmark (all the contests dealing with medicine) revealed a small treatment effect. However, the relationship between the number of total publications by applicants before and after competitions revealed that this treatment effect is the effect of the “maturation” of young scientists with a high baseline publication frequency – not of grant funding.

## Supporting Information

Figure S1 **The number of total publications of awarded and rejected applicants before (ex ante) and after (ex post) in the FRIPRO competitions (adapted from** [**10**] **). Note**. The dotted line is the regression line for the row of values for the awarded group; the solid line is the regression line for the rejected group; the dashed line is the reference line (the number of total publication ex-ante and ex-post are equal).
(TIF)

Figure S2 **The assessment of funnel plot asymmetry and potential publication bias in a collection of quasi-experimental studies.** **Note**. The effect size is the difference between the pre-post number of publications in the treatment and control groups. Egger test: bias = 1,04 (95% CI = −1,33 to 3,41), p = 0,352.
(TIF)

Table S1 **An academic degrees in Russia and the doctoral dissertation defense procedure.**
(DOCX)

Table S2 **A brief overview of the eLIBRARY.RU resource and opportunities for bibliometric analysis of Russian authors’ publication activity using this database.**
(DOCX)

Table S3 **The characteristics of quasi-experimental studies that investigated the impact of medical research/training grants on research productivity.**
(DOCX)

Table S4 **The mean number of total publications and citations per person in quasi-experimental studies included in the brief systematic review.**
(DOCX)
