# Supplementary figures and images for: The Impact of Funding through the RF President’s Grants for Young Scientists (the field – Medicine) on Research Productivity: A Quasi-Experimental Study and a Brief Systematic Review
Source: PLoS One. 2014 Jan 27;9(1):e86969. doi: 10.1371/journal.pone.0086969 (PMC3903615; doi:10.1371/journal.pone.0086969)

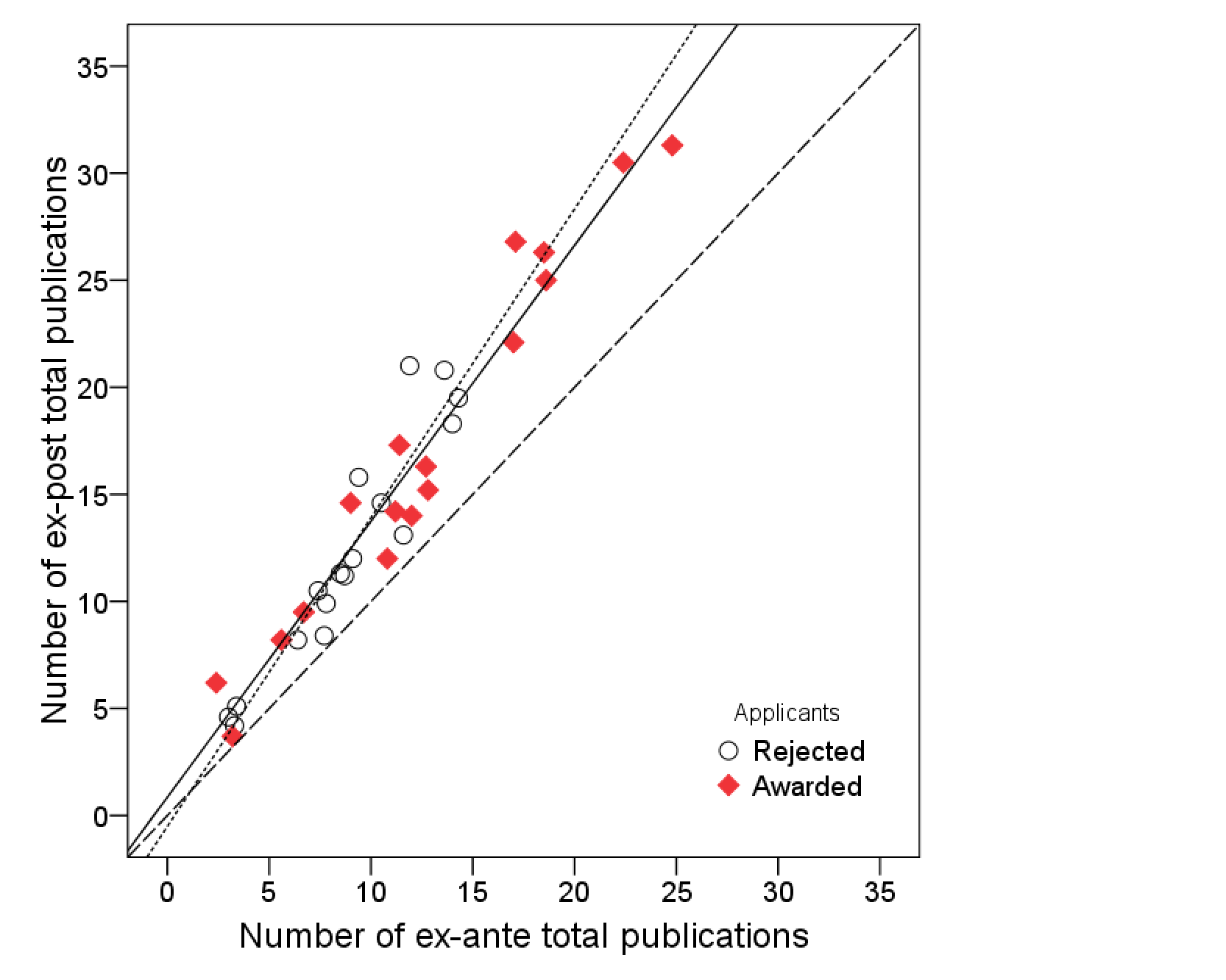

Supplement: Figure S1 — The number of total publications of awarded and rejected applicants before (ex ante) and after (ex post) in the FRIPRO competitions (adapted from [10] ). Note. The dotted line is the regression line for the row of values for the awarded group; the solid line is the regression line for the rejected group; the dashed line is the reference line (the number of total publication ex-ante and ex-post are equal). (TIF) [file pone.0086969.s001.tif]

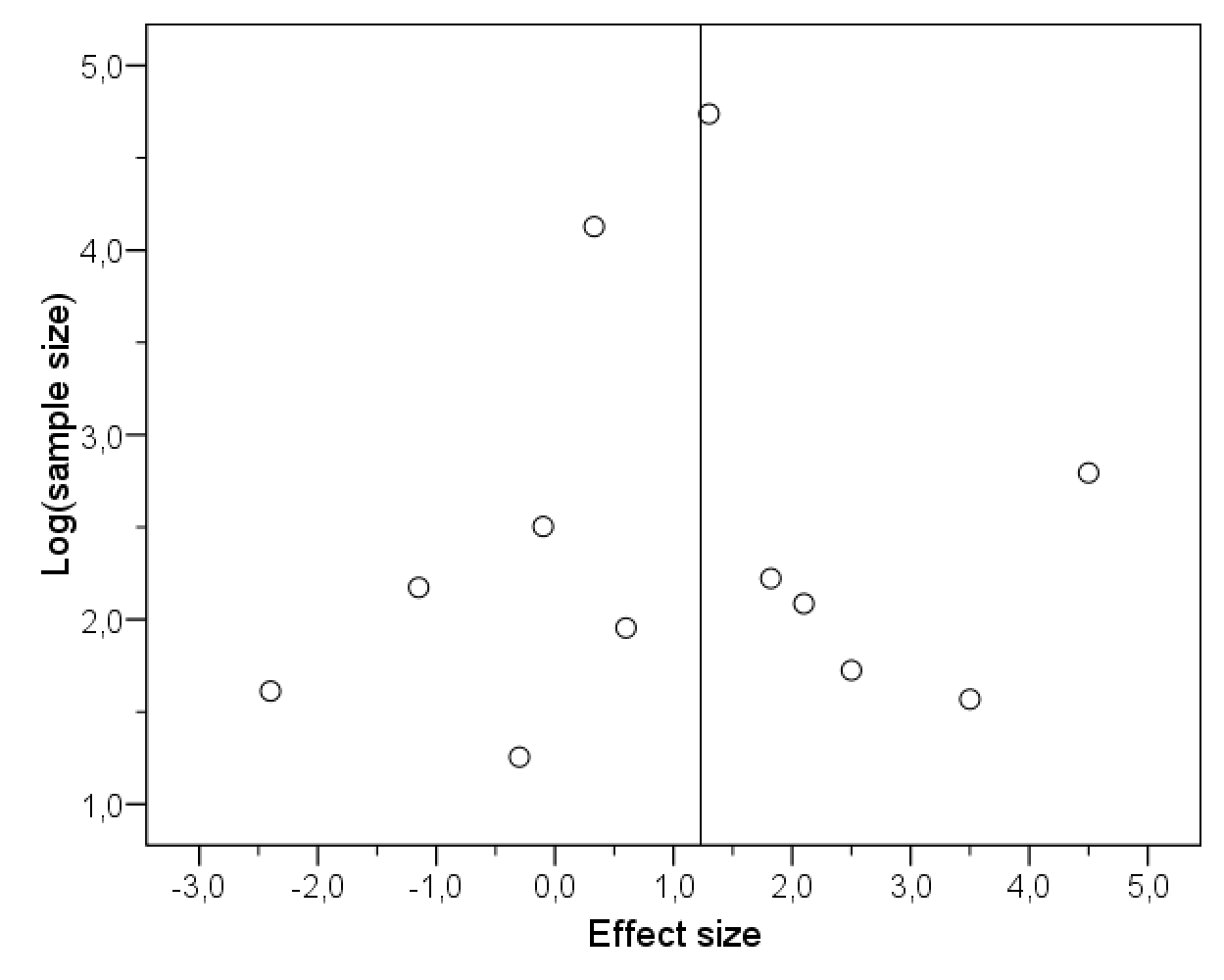

Supplement: Figure S2 — The assessment of funnel plot asymmetry and potential publication bias in a collection of quasi-experimental studies. Note. The effect size is the difference between the pre-post number of publications in the treatment and control groups. Egger test: bias = 1,04 (95% CI = −1,33 to 3,41), p = 0,352. (TIF) [file pone.0086969.s002.tif]
