# Supplementary material for: The Impact of Funding through the RF President’s Grants for Young Scientists (the field – Medicine) on Research Productivity: A Quasi-Experimental Study and a Brief Systematic Review
Source: PLoS One. 2014 Jan 27;9(1):e86969. doi: 10.1371/journal.pone.0086969 (PMC3903615; doi:10.1371/journal.pone.0086969)
Supplement: Table S1 — An academic degrees in Russia and the doctoral dissertation defense procedure. (DOCX) [file pone.0086969.s003.docx]

# Table S1. An academic degrees in Russia and the doctoral dissertation defense procedure

| **An academic degrees in Russia** |
| --- |
| Russia uses a two-tier system of awarding a post-graduate scientific (academic) degree. The first tier is Kandidat Nauk (Candidate of Sciences, CoS), the second – Doctor Nauk (Doctor of Sciences, DoS). The CoS and DoS degrees both should be considered as PhD in countries wherein there is only one doctoral degree awarded [For guidelines on the recognition of Russian qualifications in other European countries go to http://en.russia.edu.ru/edu/inostr/prizn/900/]. The CoS degree is awarded for original research that constitutes a significant contribution to a scientific field, the DoS – for original research on the basis whereof “… *there have been developed theoretical provisions which combined can count as a scientific achievement, or there has been solved a scientific problem that is of … significance, or there have been expounded scientifically well-founded … solutions the implementation whereof makes a considerable contribution to the country’s development*” [http://vak.ed.gov.ru/ru/docs/?id54=4]. |
| **A brief description of the doctoral dissertation defense procedure in Russia** |
| The defense of the doctoral dissertation is to be held at the session of the Specialized Dissertation Committee accredited by the VAK at the academic or research institutions. One is to present conclusions from three independent reviewers. At a minimum, two of those conclusions are to be oral. According to the Provision on the Procedure for the Awarding of Doctoral Scientific Degrees “*A dissertation for the Doctor of Science scientific degree is to be a scientific qualification work that contains certain theoretical statements developed on the strength of studies conducted by the author, the aggregate whereof can be qualified as a scientific achievement, or contains a solution for a scientific problem that is of political, socio-economic, cultural, or economic significance, or contains scientifically founded technical, technological, or other solutions the implementation whereof significantly contributes to the country’s development*” (Decree of the Government of the Russian Federation No.74 dated 30.01.2002 [with amendments as of 20.06.2011]; http://vak.ed.gov.ru/ru/docs/?id54=4&i54=4). If the defense of the dissertation is successful (a 66.6% majority of Specialized Dissertation Committee members votes by secret ballot; the total number of members is normally about 20, all holding the DoS degree), it is recommended and is to be approved later by the VAK. |
