# Supplementary material for: The Impact of Funding through the RF President’s Grants for Young Scientists (the field – Medicine) on Research Productivity: A Quasi-Experimental Study and a Brief Systematic Review
Source: PLoS One. 2014 Jan 27;9(1):e86969. doi: 10.1371/journal.pone.0086969 (PMC3903615; doi:10.1371/journal.pone.0086969)
Supplement: Table S2 — A brief overview of the eLIBRARY.RU resource and opportunities for bibliometric analysis of Russian authors’ publication activity using this database. (DOCX) [file pone.0086969.s004.docx]

Table S2. A brief overview of the eLIBRARY.RU resource and opportunities for bibliometric analysis of Russian authors’ publication activity using this database

| Based on information provided by the project organizers, “… *eLIBRARY.RU is the largest Russian information database for science, technology, and education, which contains abstracts and full texts of over 14 million scientific articles and publications. The eLIBRARY.RU platform offers electronic versions of over 2500 Russian scientific and technical journals*.” (<http://elibrary.ru>; data from early 2013).  Data on the total number of publications and their citations with particular researchers can be obtained in two ways. The main way is through the “Author Index” (<http://elibrary.ru/authors.asp>/), which contains detailed data on the publication activity of over 30 thousand Russian authors working in the area of medicine and health care. Authors can create their profiles in the system on their own and can edit them after registering with eLIBRARY.RU. Each profile includes information on the place of employment and publication activity (the Hirsch index, the number of self-citations, the number of co-authors, the number of publications in foreign journals, the impact-factor of journals the author’s articles were published in, etc.). However, not all Russian authors (i.e. those who have at least one publication) have their own “page” in the “Author Index”. In such cases, the eLIBRARY.RU search system (the “Full-text Search” section; <http://elibrary.ru/defaultx.asp/>) serves as an additional source of information on the number of publications and their citations. Search by the author’s last name can be specified using his/her name and patronymic, initials (one can choose from variants in the database), specialty field, and a particular time window. Compared with the “Author Index”, the eLIBRARY.RU search system provides no detailed bibliometric information on the author’s publication activity. However, even in the “Author Index” one can find only summary bibliometric data (i.e. data computed taking account of all data available in the system; for certain characteristics, it is data computed for a period of two or five years), and this data cannot be examined by years.  The issue of the presence of international publications by Russian authors in the eLIBRARY.RU database warrants separate clarification. Below is an excerpt from an interview (published on July 20, 2010) with the developers of the eLIBRARY.RU database Gennady Yeremenko and Victor Glukhov [Ref 22]: “*Thompson Reuters presented us with the opportunity to make real-time queries with Web of Science and show the number of citations of a particular publication from the Russian Science Citation Index (RSCI). … We acquired from Elsevier the full array of publications by Russian authors, i.e. articles containing “Russia” in the addresses, spanning the period of 1996 through 2011, as well as all articles from Scopus which reference these publications. They are currently in the stage of being processed and integrated with RSCI data. … The first results, which are based on the joint data of Scopus and the RSCI, will come out as early as the end of July, and we are planning to be done with tying these integral data to Russian authors and institutions by September*.” Thus, there are grounds to believe that the bibliometric analysis conducted in early 2013 using the eLIBRARY.RU database, provides the same volume of information in relation to international publications by Russian authors as WoS and Scopus. |
| --- |
