# Supplementary material for: The Impact of Funding through the RF President’s Grants for Young Scientists (the field – Medicine) on Research Productivity: A Quasi-Experimental Study and a Brief Systematic Review
Source: PLoS One. 2014 Jan 27;9(1):e86969. doi: 10.1371/journal.pone.0086969 (PMC3903615; doi:10.1371/journal.pone.0086969)
Supplement: Table S3 — The characteristics of quasi-experimental studies that investigated the impact of medical research/training grants on research productivity. (DOCX) [file pone.0086969.s005.docx]

Table S3. The characteristics of quasi-experimental studies that investigated the impact of medical research/training grants on research productivity

| **Foundation (author)** | **Country** | **Fields** | **Date of competition, years** | **Type of grant** | **Size of grant** | **Source of bibliometric data** | **Type of publication** | **Time window for bibliometric analyses (before/after competition), years** |
| --- | --- | --- | --- | --- | --- | --- | --- | --- |
| AAFP Joint Grant Awards Program (Mahoney M.C., 2006 [18]) | USA | Family medicine | 1996-2000 | research | <25 000 $ (total per year) | PubMed, survey | Peer-reviewed publications | 5/5 |
| Emmy Noether Programme (Böhmer S., 2008 [2])**§** | Germany | Medicine, microbiology, virology, immunology, neuroscience | 1999-2006 | research | Not available | WoS, survey | WoS cited publications | 4*/4* |
| Danish Council for Independent Research, 2011 (first time applicants) [5]**§** | Denmark | Medical sciences | 2002-2004 | research | 1.2 million DKK (mean, total) | Scopus | Published in journals, proceedings, and books | 4**/4*** |
| NIH post-doctoral fellowships (F32) (Jacob B., 2011 [6]) | USA | Health-related sciences (biological – 82%) | 1980-2000 | training | 50 000 $ (mean per year) | WoS | WoS cited publications | 5/5 |
| NIH standard research grants (R01s) (Jacob B., 2011 [7]) | USA | Health-related sciences | 1980-2000 | research | 1.7 million $ (mean, total) | WoS | WoS cited publications | 5/5 |
| FRIPRO – Research Council of Norway (Langfeldt L., 2012 [10]) | Norway | Biomedicine, Pharmacology & Toxicology, Clinical sciences, Social medicine and Epidemiology, Psychology | 2005-2007 | research, training | 3.4 million NOK (mean, total) | WoS | WoS cited publications | 5/5 |

**Note**: * – the year of the competition was held was not taken into account in the analysis of bibliometric data; **– including the year the competition was held; *** – a time window of up to two years following the application date. **§** – the articles were translated from the national language (German, Danish) into English using an online translator (<https://translate.google.ru>).
