# Supplementary material for: The Impact of Funding through the RF President’s Grants for Young Scientists (the field – Medicine) on Research Productivity: A Quasi-Experimental Study and a Brief Systematic Review
Source: PLoS One. 2014 Jan 27;9(1):e86969. doi: 10.1371/journal.pone.0086969 (PMC3903615; doi:10.1371/journal.pone.0086969)
Supplement: Table S4 — The mean number of total publications and citations per person in quasi-experimental studies included in the brief systematic review. (DOCX) [file pone.0086969.s006.docx]

Table S4. The mean number of total publications and citations per person in quasi-experimental studies included in the brief systematic review

| **Foundation (author)** | **Applicants** | **N, abs.** | **Number of total articles, mean (SD*)** | | | **Number of total citations, mean** | |
| --- | --- | --- | --- | --- | --- | --- | --- |
|  |  |  | **before** | **after** | **difference** | **before** | **after** |
| AAFP Joint Grant Awards Program (Mahoney M.C., 2006 [18]) | Awarded | 17 | 4.9 (5.40) | 9.1 (6.60) | 4.2 **(3.96)** | --- | --- |
|  | Rejected | 36 | 2.7 (4.20) | 4.4 (4.20) | 1.7 **(2.66)** | --- | --- |
| Emmy Noether Programme (Böhmer S., 2008 [2])** | Awarded | 68 | 10.1 **(9.87)** | 14.7 **(12.24)** | 4.7 **(7.34)** | 46.1 | 36.9 |
|  | Rejected | 99 | 12.6 **(15.20)** | 15.5 **(15.91)** | 2.8 **(9.86)** | 35.2 | 33.0 |
| Danish Council for Independent Research, 2011 [5] | Awarded | 315 | 22.8 **(20.92)** | 29.8 **(23.30)** | 7.0 **(14.16)** | 169.0 | 260.1 |
|  | Rejected | 307 | 14.2 **(16.64)** | 16.7 **(16.76)** | 2.5 **(10.56)** | 62.2 | 83.5 |
| NIH post-doctoral fellowships (F32) (Jacob B., 2011 [6]) | Awarded | 6176 | 3.2 **(3.02)** | 5.4 **(4.32)** | 2.2 **(2.63)** | --- | 239.8 |
|  | Rejected | 7250 | 2.7 (3.30) | 4.6 (4.50) | 1.9 **(2.74)** | --- | 200.4 |
| NIH standard research grants (R01s) (Jacob B., 2011 [7]) | Awarded | 39294 | 16.8 **(14.29)** | 19.4 **(14.14)** | 2.6 **(8.99)** | --- | 678.7 |
|  | Rejected | 15447 | 15.4 **(16.72)** | 16.7 **(15.58)** | 1.3 **(10.27)** | --- | 553.0 |
| FRIPRO – Research Council of Norway (Langfeldt L., 2012 [10]): Biomedicine | Awarded | 72 | 12.8 **(12.40)** | 15.2 **(12.60)** | 2.4 **(7.91)** | --- | --- |
|  | Rejected | 247 | 8.7 **(10.47)** | 11.2 **(11.50)** | 2.5 **(7.02)** | --- | --- |
| FRIPRO – Research Council of Norway (Langfeldt L., 2012 [10]): Pharmacology & Toxicology | Awarded | 4 | 10.8 **(11.12)** | 12.0 **(10.61)** | 1.2 **(6.89)** | --- | --- |
|  | Rejected | 14 | 11.6 **(14.53)** | 13.1 **(14.08)** | 1.5 **(9.06)** | --- | --- |
| FRIPRO – Research Council of Norway (Langfeldt L., 2012 [10]): Clinical sciences | Awarded | 27 | 18.6 **(18.03)** | 25.0 **(20.63)** | 6.4 **(12.47)** | --- | --- |
|  | Rejected | 95 | 14.0 **(16.78)** | 18.3 **(18.69)** | 4.3 **(11.36)** | --- | --- |
| FRIPRO – Research Council of Norway (Langfeldt L., 2012 [10]): Social medicine and Epidemiology | Awarded | 20 | 17.1 **(16.73)** | 26.8 **(22.16)** | 9.7 **(13.33)** | --- | --- |
|  | Rejected | 70 | 11.9 **(14.45)** | 21.0 **(21.43)** | 9.1 **(13.14)** | --- | --- |
| FRIPRO – Research Council of Norway (Langfeldt L., 2012 [10]): Psychology | Awarded | 8 | 9.0 **(9.23)** | 14.6 **(12.63)** | 5.6 **(7.63)** | --- | --- |
|  | Rejected | 29 | 7.8 **(9.82)** | 9.9 **(10.64)** | 2.1 **(6.52)** | --- | --- |
| RF President’s Grant for Young Scientists (CoS’s) (Saygitov R., 2013) | Awarded | 24 | 5.3 (4.60) | 7.8 (8.90) | 2.5 (6.90) | 2.3 | 9.4 |
|  | Rejected | 125 | 4.9 (5.70) | 8.5 (9.10) | 3.6 (7.26) | 3.1 | 8.1 |
| RF President’s Grant for Young Scientists (DoS’s) (Saygitov R., 2013) | Awarded | 22 | 11.2 (11.20) | 20.7 (17.50) | 9.5 (9.04) | 5.8 | 12.6 |
|  | Rejected | 19 | 16.3 (19.50) | 28.2 (30.80) | 11.9 (18.20) | 8.8 | 19.0 |

**Note.** * The calculated standard deviation is in bold. The steps for calculating the missing values of SD are described below. ** In the article, the numerical data were presented graphically. It’s digitized using GetData Graph Digitizer v.2.25.0.32 (<http://getdata-graph-digitizer.com/>).

**Imputing a change-from-baseline standard deviation**

In randomized studies, to compare the results of interventions and to combine these results in the meta-analysis we just need the values of the final measurements (the mean and SD in the experimental and control groups). When it comes to non-randomized controlled studies and the meta-analysis of their results, it is recommended to use the change-from-baseline mean and SD in each interventional group [Ref 1]. If this data is not available, the mean change can be obtained by simply subtracting the baseline mean from the final mean. To calculate the unreported values of the change-from-baseline SD for each of the interventional groups we will need the baseline and final values of the SD and the correlation coefficient (assessment of the similarity between the baseline and final measurements) [Ref 1]. However, the values of these variables are often unavailable. One of the ways to impute the missing values of the SD is a linear regression of log(SD) on log(mean); the two are strong correlated variables [Ref 2]. The value of the correlation coefficient (if unreported) “*might be imputed from another study … it might be imputed from elsewhere, or it might be hypothesized based on reasoned argument*.” [Ref 1].

*References:*

1. Higgins JPT, Green S (editors). Cochrane Handbook for Systematic Reviews of Interventions Version 5.1.0 [updated March 2011]. The Cochrane Collaboration, 2011. Available: [www.cochrane-handbook.org](http://www.cochrane-handbook.org). Accessed 6 August 2013.
2. Marinho VC, Higgins JP, Sheiham A, Logan S (2003). Fluoride toothpastes for preventing dental caries in children and adolescents. Cochrane Database Syst Rev (1): CD002278.

In the brief systematic review, the baseline and final values of the SD are known for Jacob B. et al. [6] (for the control group only), Mahoney M.C. et al. [18] and Russian studies; 14 pairs of “mean-SD” values in total (see Table S4). From the analysis of the 14 available pairs of “mean-SD” values I derived a regression equation log(SD) = 0.388 +0.954*log(Mean) - 0.222*Applicants - 0.147*Time window - 0.019*log(Number of applicants), where “log(Mean)” is the log transformed values of the mean (the number of total articles), “Applicants” – dummy variables (where 0 – rejected, 1 – awarded), “Time window” – also dummy variables (where 0 – values of the mean and SD for the period “before”, 1 – for the period “after”), log(Number of applicants) – the log transformed number of applicants. The linear model is explained through the high percentage of the dependent variable variation (R squared = 95%). This equation was used to estimate missing SD. The logarithmic form of the predicted values of the SD was converted to exponential form and was used in this form to calculate the change-from-baseline SD. If the values of the SD were known, the predicted values of the next analysis were excluded. The value of the correlation coefficient used for calculating the change-from-baseline SD, was 0.80 (both for the experimental and control groups). For example, in the Russian study the correlation coefficient of the number of total articles before and after competitions was 0.81 (calculated for all applicants, n = 190).

The validity of the imputing technique was checked through the example of combining the data from two competitions in the Russian study. It was shown that the difference in the mean change in the number of total articles in using the known values of the SD in the meta-analysis produced a pooled estimate (weighted mean difference) which equaled -1.28 (95% CI -4.16 to 1.60). In combining the data using the calculated values of the SD, the pooled estimate of the effect was -1.23 (95% CI -3.72 to 1.26). Thus, the confidence interval for the weighted mean difference obtained using the calculated values of the SD was already narrower than the confidence interval calculated based on the known values of the SD, by 14% ((4.98/5.76)*100%).
